# Impact of anesthetic drugs and modalities on the postoperative outcomes in cancer patients: a literature review

**DOI:** 10.3389/fmed.2026.1735369

**Published:** 2026-03-31

**Authors:** Yangyang Ge, Xiaochen Qi, Tianzhi Chang, Yong Luan

**Affiliations:** First Affiliated Hospital, Dalian Medical University, Dalian, China

**Keywords:** anesthesia, anesthetic drug, cancer prognosis, cancer prognostics, cancer therapy, immunomodulation, postoperative management

## Abstract

**Background:**

Growing evidence suggests that perioperative anesthesia management may influence long-term oncologic outcomes. This review synthesizes the existing evidence on the impact of anesthetic drugs and modalities on postoperative immunity, recurrence, and survival in cancer patients.

**Methods:**

A systematic literature search was conducted in PubMed, Embase, Cochrane Library, and Web of Science from January 2000 to October 2025. Keywords included combinations of “anesthesia,” “anesthetic,” “cancer,” “oncology,” “postoperative,” “recurrence,” “immunity,” and “survival.” Clinical trials, cohort studies, and meta-analyses were included. Case reports and non-English studies were excluded.

**Results:**

Anesthetic choices exert multidimensional effects. Total intravenous anesthesia (TIVA) with propofol and regional anesthesia (RA) techniques are associated with better preservation of natural killer (NK) cell and T-lymphocyte function compared to volatile anesthetics and high-dose opioids. Opioids, particularly morphine, demonstrate dose-dependent immunosuppression (15–30% NK cell reduction). Meta-analyses indicate RA may reduce recurrence risk (OR = 0.82, *p* < 0.01). However, conflicting evidence exists, with large retrospective and some randomized controlled trials (RCTs) showing no significant survival difference between TIVA and volatile anesthesia. Technological advances like circulating tumor DNA (ctDNA) monitoring and AI-driven analgesic algorithms promise personalized management.

**Conclusion:**

While preclinical and clinical data suggest that anesthetic strategy can modulate cancer-related outcomes, the evidence is heterogeneous. The observed benefits may be context-dependent, influenced by tumor type, surgical stress, and patient factors. There is an urgent need for large-scale, prospective RCTs with standardized endpoints to establish causal relationships and inform evidence-based, personalized anesthesia protocols for oncology patients.

## 1 Introduction

Cancer remains a leading cause of global morbidity and mortality, with surgical resection constituting a cornerstone of curative treatment for solid tumors. While anesthesia is indispensable for enabling these complex procedures, emerging evidence suggests that the choice of anesthetic modalities and pharmacologic agents extends far beyond intraoperative comfort and safety. A growing body of research indicates that perioperative anesthesia management may significantly influence critical postoperative outcomes in oncology patients, including immune function, inflammatory responses, pain control, recovery quality, complication rates, and, crucially, long-term oncologic outcomes such as cancer recurrence and survival.

The perioperative period represents a vulnerable window for cancer patients. Surgical stress itself can induce immunosuppression, neuroendocrine activation, and release of pro-inflammatory cytokines, potentially creating a microenvironment conducive to residual tumor cell survival, proliferation, and metastasis. Anesthetic techniques and drugs, interacting with these complex pathways, may either mitigate or exacerbate these effects. Key mechanisms under investigation include the modulation of natural killer (NK) cell activity, T-lymphocyte function, hypoxia-inducible factors (HIFs), systemic inflammatory responses, and stress hormone release (e.g., cortisol, catecholamines). Furthermore, the adequacy of postoperative pain management directly impacts recovery, sleep quality, and patient wellbeing.

Despite its potential significance, the relationship between anesthesia and postoperative outcomes in oncology is complex and often characterized by conflicting clinical findings. Questions persist regarding the comparative effects of total intravenous anesthesia (TIVA) versus volatile anesthetics, the immunomodulatory impact of opioids versus opioid-sparing techniques like regional anesthesia, and the long-term survival implications of specific anesthetic choices. The development of novel anesthetic agents, advanced monitoring technologies, and personalized analgesic strategies further adds layers of complexity and opportunity to perioperative care.

**Figure 1:**
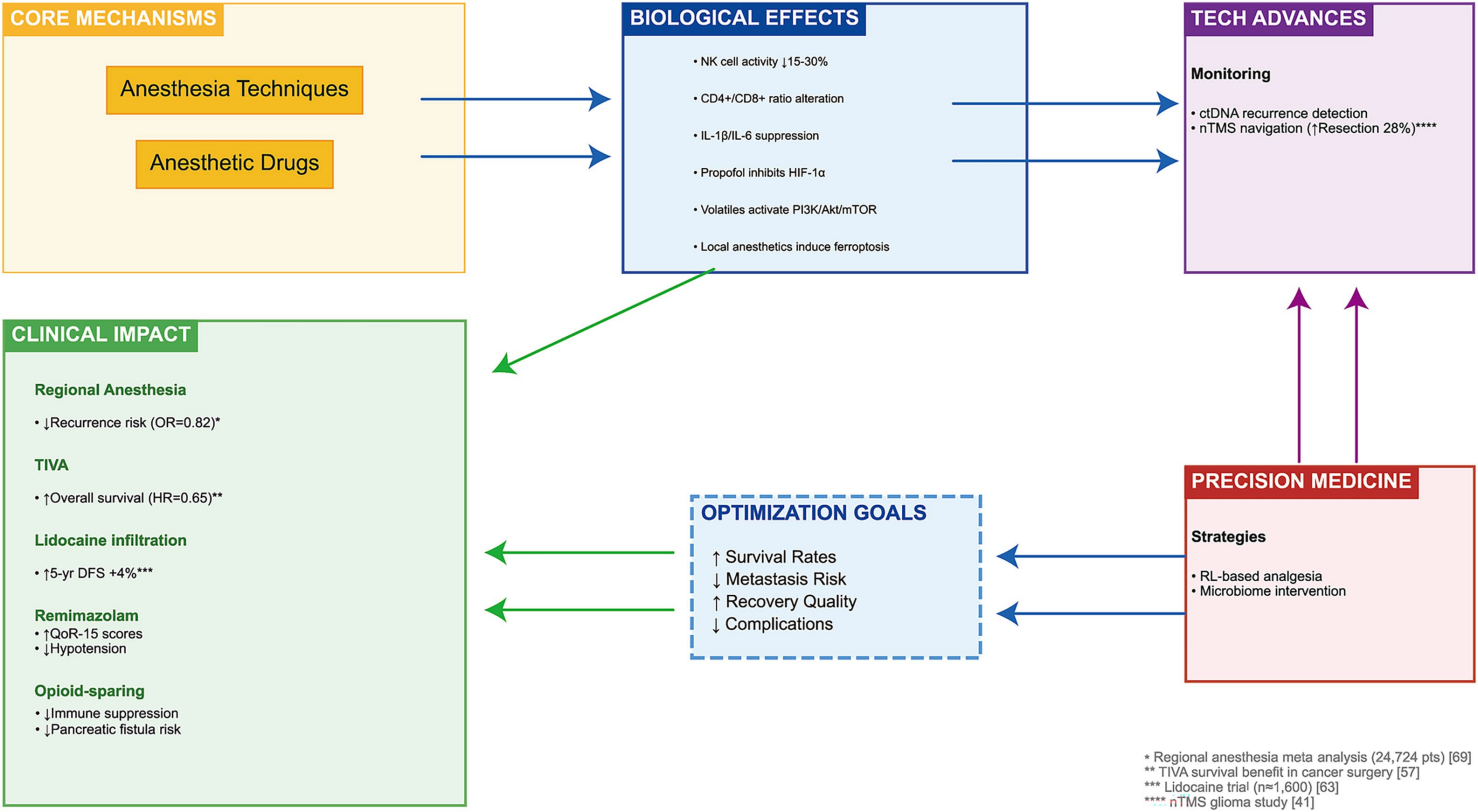
The effect of anesthetics on tumor patients in clinical treatment.

This review aims to provide a comprehensive synthesis of the current state of knowledge on the effects of anesthesia modalities and drugs on the postoperative period in oncology patients. It systematically examines the evidence across multiple dimensions: fundamental biological mechanisms (immune, inflammatory, stress response), clinical outcomes (recovery, complications, pain control), technological advancements (new drugs, monitoring, optimization strategies), historical evolution of practices, current standards and controversies, and future directions for personalized care and research. By integrating findings from basic science, clinical trials, and meta-analyses, this review seeks to elucidate the multifaceted role of anesthesia in the oncologic surgical journey and provide a foundation for optimizing perioperative management to improve both immediate recovery and long-term prognosis (Figure 1).

## 2 Methods

### 2.1 Search strategy

A systematic literature search was conducted in PubMed, Embase, Cochrane Library, and Web of Science from January 2000 to October 2025. Keywords included: (“anesthesia” OR “anesthetic”) AND (“cancer” OR “oncology”) AND (“postoperative” OR “recurrence” OR “immunity” OR “survival”).

### 2.2 Inclusion and exclusion criteria

Inclusion: clinical trials, cohort studies, meta-analyses, and reviews focusing on anesthesia and cancer outcomes.

Exclusion: case reports, non-English studies, non-human studies.

### 2.3 Study selection and data extraction

Two authors (YG and TC) independently screened titles and abstracts. Full texts of potentially eligible records were then retrieved and independently assessed for inclusion by the same two authors. Any discrepancies regarding study eligibility were resolved through discussion with a third author (XQ) until a consensus was reached.

Data extraction was performed independently by two authors (YG and TC) using a predefined and standardized data extraction form. The following information was systematically collected from each included study: (1) study characteristics (first author, year of publication, country, study design, sample size, follow-up duration); (2) patient population (cancer type, stage, surgical procedure); (3) anesthetic intervention (type of anesthesia e.g., TIVA, volatile, regional e.g., TIVA, volatile, regional, specific anesthetic drugs used, details of analgesic regimen); (4) comparator (alternative anesthetic modality or drug); (5) outcomes measured (primary and secondary outcomes, including immune function markers e.g., NK cellactivity, cytokinelevels e.g., NK cellactivity, cytokinelevels, postoperative complications, recurrence rates, and survival data e.g.,overallsurvival,recurrence−freesurvivale.g.,overallsurvival,recurrence−freesurvival); and (6) main findings and conclusions. Extracted data were cross-checked for accuracy, and any disagreements were resolved by consensus.

### 2.4 Quality and risk of bias assessment

To assess the methodological quality and risk of bias of the included studies, a formal appraisal was conducted independently by two reviewers (TC and XQ). For randomized controlled trials (RCTs), the Cochrane Risk of Bias tool (RoB 2) was used to evaluate bias across domains including randomization process, deviations from intended interventions, missing outcome data, measurement of the outcome, and selection of the reported result. For observational studies (cohort and case–control studies), the Newcastle-Ottawa Scale (NOS) was employed. The NOS assesses studies based on three broad categories: selection of study groups, comparability of groups, and ascertainment of outcome/exposure. Studies were assigned a star rating, with a higher number of stars indicating higher quality. Any disagreements in the quality assessment were resolved through discussion or consultation with a senior author (YL). The results of this quality appraisal were considered when interpreting the findings and drawing conclusions, particularly when weighing evidence from studies with varying methodological rigor.

### 2.5 Summary of included studies

**Figure 2:**
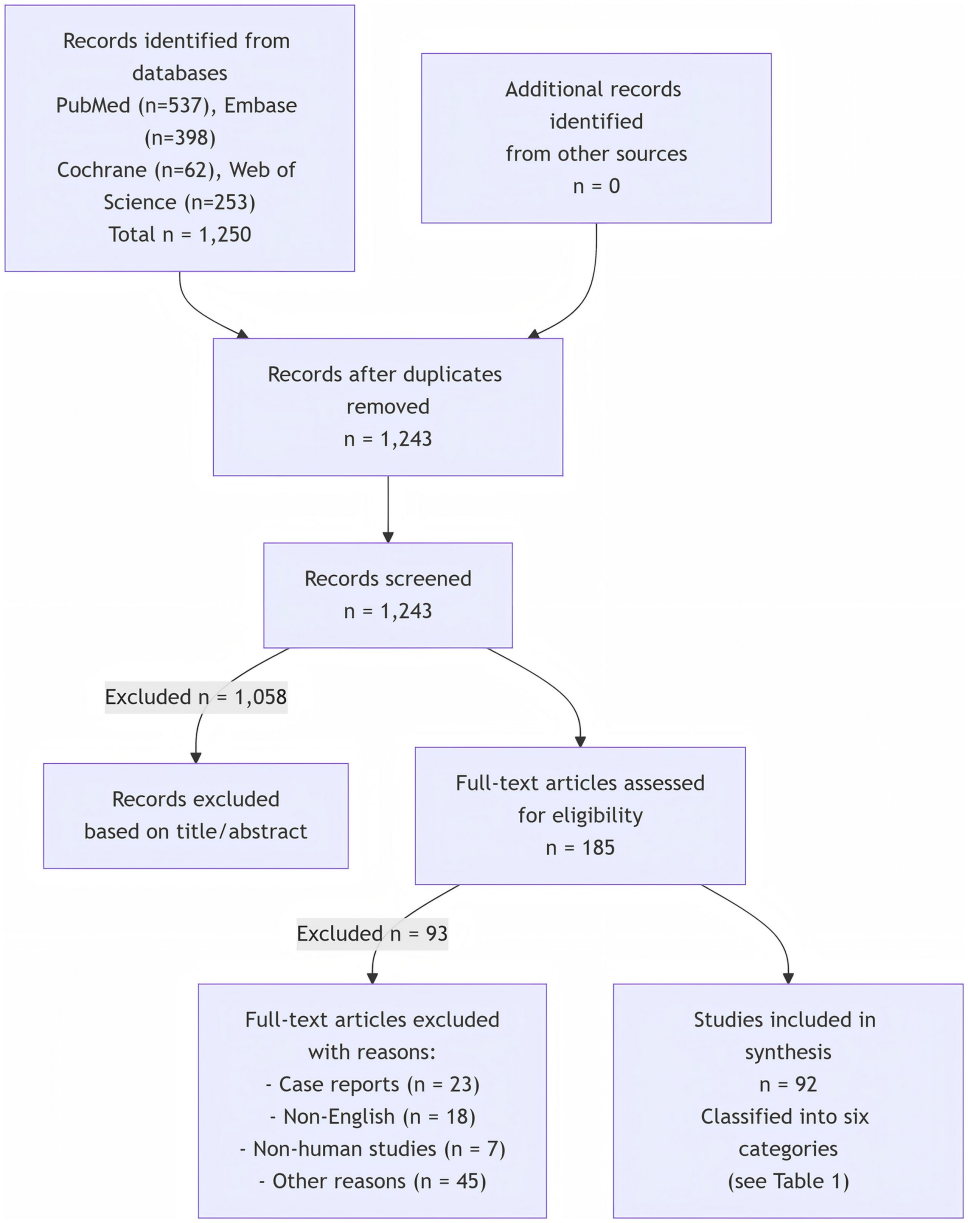
Literature screening process.

Initial search yielded 1,250 records; 185 full-text articles were assessed; 92 studies met inclusion criteria (Figure 2). Considering the suitability to the research direction of this review, a total of 75 articles were included, including research articles and review articles. All the studies were classified into six different research directions (Table 1).

**Table 1:** The postoperative effects and mechanisms of different anesthetics on cancer patients.

Drug class	Representative drug(s)	Mechanism of action	Impact on postoperative recovery	Related studies
Propofol	Propofol	- Inhibits glycolysis pathway, reduces adhesion between tumor cells and endothelial cells	- Reduces thyroid cancer recurrence rate (RR = 0.38)	(22, 45, 51, 62)
		- Activates tumor cell apoptosis pathways (caspase cascade)	- Improves postoperative sleep in elderly patients (extends by 29 min)	
		- Upregulates E-cadherin to inhibit migration	- Reduces need for postoperative mechanical ventilation	
		- Reduces pro-inflammatory cytokine release (e.g., NF-κB), protects T-cell function		
		- Reduces opioid consumption		
Remimazolam	Remimazolam	- Enhances levels of NK cells, CD3+, CD4 + cells	- Improves postoperative recovery quality (↑QoR-15 score)	(5, 14, 15)
		- Short-acting metabolism reduces perioperative stress response	- Reduces intraoperative hypotension/bradycardia incidence	
Volatile anesthetics	Sevoflurane/desflurane	- Upregulates HIF-1α (promotes tumor invasive phenotype)	- Reduces pulmonary complications after esophageal cancer surgery (36.5% vs. 47.5%)	(13, 18, 61)
		- Downregulates Natural Killer (NK) cell cytotoxicity	- No significant survival difference vs. Propofol	
		- Maintains PEEP to reduce alveolar collapse, lowers IL-6 (↓40%)		
Opioids	• Morphine	- Suppresses NK cell activity via μ-receptors (↓15–30%)	- Increases pancreatic fistula risk (↑13% per 100 mg MME dose increase)	(31, 36, 42)
		- Inhibits T-lymphocyte proliferation	- Associated with immunosuppression-related fatigue	
	• Fentanyl	- High doses (>25 μg/kg) inhibit lymphocyte proliferation	- High doses (>28.2 μg/kg) positively correlate with NSCLC survival	(47, 55)
			- Increases postoperative pulmonary complication risk	
	• Tramadol	- Weak μ-receptor agonist + monoamine reuptake inhibitor; preserves immune activity	- Reduces postoperative infection risk	(17, 33)
			- Attenuates immunosuppression	
Dexmedetomidine	Dexmedetomidine	- Suppresses perioperative inflammatory response	- Reduces HR index and inflammatory cytokine levels (↓IL-6, etc.)	(20)
		- Stabilizes blood gas microcirculation and pulmonary function		
Local anesthetics	• Lidocaine	- Induces tumor cell ferroptosis/apoptosis (inhibits PI3K/Akt pathway)	- Improves 5-year breast cancer survival (87% vs. 83%)	(52, 62, 63)
		- Enhances anti-tumor immunity (preserves NK cell activity)	- Reduces serum S100A8/A9 (↓41%, suppresses metastasis)	
	• Ropivacaine	- Blocks sympathetic excitation, reduces stress hormone release	- Reduces intraoperative opioid use in thyroid cancer (≥50%)	(48, 49)
			- Improves postoperative recovery index and pain scores	

## 3 Mechanisms of anesthesia-immune interaction

The selection of anesthesia for oncological patients necessitates a comprehensive consideration of multiple factors. The type and location of the surgical procedure serve as crucial determinants. For instance, mediastinoscopy, bronchoscopy, and thoracoscopy, which are invasive techniques employed for the staging of lung cancer tumors, present distinct challenges in anesthesia administration. Cata et al. (1) Moreover, the patient’s individual condition cannot be overlooked. With the increasing prevalence of obese cancer patients, specific guidelines need to be formulated to optimize the surgical process (2). Diverse anesthesia modalities yield different prognoses for patients. A cohort study involving patients with lung, breast, or esophageal cancer demonstrated that the overall survival rate of the total intravenous anesthesia (TIVA) group was significantly higher than that of the inhalation - intravenous anesthesia group (*p* = 0.042). However, there was no significant difference in the recurrence - free survival rate between the two groups (*p* = 0.296) (3). Another retrospective analysis of gastrointestinal cancer surgeries indicated that there were no statistically significant differences in the postoperative complication rates, 3—year overall survival, and progression - free survival between patients who underwent propofol total intravenous anesthesia and those who received inhalational anesthesia (*p* = 0.9217, *p* = 0.9918, and *p* = 0.9981) (4). Anesthesia modality exerts a significant impact on the postoperative immune function of oncological patients. Taking laparoscopic colorectal cancer surgery as an example, patients who received remazolam general anesthesia exhibited significantly higher levels of natural killer (NK) cells, CD3 +, and CD4 + cells immediately after surgery and 24 h post—surgery compared to those in the propofol group. This finding suggests that remazolam anesthesia better preserves perioperative immune function (5). Different anesthesia modalities influence immune function through diverse mechanisms. In total hip arthroplasty, the general anesthesia group showed significantly lower *in vitro* IL - 1β cytokine production capacity than the spinal anesthesia group immediately after surgery (*p* = 0.02), and significantly lower IL - 1β and IL - 6 levels were also observed at the end of the surgery (*p* = 0.002 and *p* = 0.02). This indicates that general anesthesia transiently affects innate immune function (6). Additionally, general anesthetics (e.g., propofol) may indirectly attenuate the inflammatory response capacity of monocyte—macrophages by inhibiting the transcription factor STAT3 signaling pathway, thereby further affecting the innate immune response (7). In cervical cancer surgery, the combination of general anesthesia and epidural block can reduce the patients’ postoperative stress response and protect immune function. During the anesthesia maintenance period and at all postoperative time points, the levels of GH, PRL, and Cor, the ratio of Th2 and Treg cells, and the levels of IL - 4 and TGF - β1 in this group were significantly lower than those in the general anesthesia group (*p* < 0.05), while the ratio of Th1 cells, the Th1/Th2 cell ratio, IFN - *γ* level, and IFN - γ/IL - 4 ratio were higher (*P* < < 0.05) (8). Notably, epidural blockade attenuates the release of glucocorticoids (e.g., Cor) and catecholamines due to surgical stress by blocking sympathetic excitation, which reduces their inhibitory effects on T-cell differentiation, and especially facilitates the maintenance of Th1-type immune responses (8). Effective postoperative pain management is crucial for oncology patients. Intelligent analgesic management system can lower the occurrence of moderate to severe postoperative pain and emesis, improving patient satisfaction outcomes Studies have shown that patients using this system had a significantly lower incidence of moderate-to-severe pain (*p* = 0.039), a lower incidence of nausea and vomiting (*p* = 0.037), and a higher level of patient satisfaction (*p* < 0.001) than those in the traditional patient-controlled analgesia group (9). The core technology of this system lies in the dynamic dose optimization algorithm based on reinforcement learning (*α* = 0.2), which adjusts drug combinations through real-time analysis of pain gene expression (e.g., the COMT rs4680 locus) to achieve individualized analgesia (↑52% accuracy of pain control) (10).

Different anesthetic modalities combined with multimodal analgesia have different impacts on patients’ postoperative pain. Studies on colorectal cancer patients showed that patients in the general anesthesia combined with epidural anesthesia and multimodal analgesia group had shorter recovery times for postoperative positioning, extubation, eye opening, and spontaneous breathing (*p* < 0.05), lower VAS scores at 24 and 48 h after surgery, higher Ramany scores at 6 and 12 h, and better cognitive function at 24, 48, and 72 h (*p* < 0.05). This suggests that this anesthesia can achieve better postoperative analgesic and sedative effects and promote the recovery of patients (11).

## 4 Clinical outcomes by cancer type

Care is needed in the selection and dose adjustment of anesthetic drugs for oncology patients. A retrospective cohort study of patients undergoing surgery for non-small cell lung cancer showed that there was no significant difference between patients receiving total intravenous anesthesia (TIVA) versus volatile anesthesia in terms of cancer-specific survival (*p* = 0.802) or overall survival (*p* = 0.736), and there was also no significant association between volatile anesthesia exposure and cancer-specific survival (univariate *p* = 0.357, multi variable *p* = 0.673) (12). Different anesthetic drugs have different mechanisms of action on tumor cells. Some volatile anesthetics upregulate hypoxia-inducible factor - 1α (HIF - 1α), whose activity is associated with a more invasive phenotype and poorer clinical prognosis, and may promote tumor recurrence, whereas other anesthetics may downregulate HIFs or have no significant effect, and are better suited for cancer surgery (13). Anesthetic agents have a wide range of effects on postoperative recovery in oncology patients. Particularly in the oncological setting, the drug properties of remazolam such as short-acting nature and rapid metabolism may help to reduce perioperative stress, thereby indirectly supporting immune system function, which may have additional benefits for cancer patients (14). In laparoscopic colorectal cancer surgery, remazolam anesthesia resulted in higher QoR-15 scores at 24 and 72 h postoperatively compared with propofol, suggesting that it improves the quality of postoperative recovery and that this improvement is not limited to subjective scores, but may extend to postoperative pain management and early mobility, accelerating the overall recovery process (15). And the incidence of intraoperative hypotension, bradycardia, and the use of vasopressor medications were lower in this group (5). The effect of opioids on immune function in cancer patients is of great concern. Although they may relieve intraoperative or chronic cancer pain, their effect on immune function is unclear. One study analyzed cancer patients using different opioids and identified distinct immune function effects among different opioids, such as morphine, oxycodone, fentanyl, and tramadol (16). Among them, tramadol combines weak opioid agonist and monoamine reuptake inhibitory properties, and its clinical data on reducing the risk of postoperative infections correlates with immunoprotective effects, providing potential advantages for cancer patients (17).

The selection and use of anesthetic drugs has a marked impact on postoperative complications in oncology patients. Volatile anesthetics reduce alveolar atrophy by maintaining optimal positive end-expiratory pressure (PEEP), as well as significantly lowering levels of the pro-inflammatory factor IL-6 (by an average of 40%) and attenuating surgery-associated inflammatory injury to lung tissue (18). In minimally invasive esophageal cancer surgery, patients receiving volatile anesthesia (sevoflurane or desflurane) exhibited a significantly lower 7-day postoperative pulmonary complication rate compared to the propofol intravenous anesthesia group [36.5% vs. 47.5%, prevalence ratio (PR) 0.63, 95% CI: 0.44–0.91, *p* = 0.013], with less severe complications (*p* = 0.035) (12). Deep sedation with propofol predisposes to central respiratory depression, which was shown in the study to triple the need for postoperative mechanical ventilation in its group (17.2% vs. 8.6%) and prolong ICU stay (19). Different anesthetic drugs affect the inflammatory response differently. Based on lung cancer surgery, compared with anesthesia with propofol and remifentanil alone, anesthesia induction with combined dexmedetomidine significantly reduced patients’ perioperative inflammatory response, stabilized patients’ blood gas microcirculation and lung function, and HR indexes of the intervention group showed significant reductions compared to the control group at both T1 and T2 time points (*p* < 0.001), and the levels of inflammatory factors were also lower (*p* < 0.05) (20).

## 5 Technological and pharmacologic advances

Novel anesthetic agents are indispensable for the postoperative management of oncology patients. Research has indicated that diverse anesthetic modalities and drugs can exert a significant influence on tumor recurrence and metastatic dissemination following surgery. Anesthetic drugs are not merely intended to offer intraoperative analgesia and sedation; their effects on tumor growth and metastasis are also garnering increasing attention (21). For instance, propofol, a frequently employed intravenous anesthetic agent, has been demonstrated to impede tumor metastasis by inhibiting the glycolytic pathway, thereby reducing the adhesion of tumor cells to endothelial cells (22). Moreover, innovative hemostatic drugs targeting the risk of perioperative bleeding are also under development. These drugs can enhance the safety of chronic antithrombotic therapy in acute and trauma surger (23). The utilization of these novel drugs not only enriches the therapeutic choices available to anesthesiologists but also provides more options for postoperative management. In the case of colorectal cancer patients, the combination of general anesthesia with epidural anesthesia and multimodal analgesia can effectively alleviate postoperative pain and facilitate patient recovery. In the study, patients in this group exhibited shorter postoperative recovery times, lower Visual Analogue Scale (VAS) scores, and alleviated inflammatory stress, along with improved immune homeostasis (11). In hepatocellular carcinoma interventional therapy, the rational selection of anesthesia methods and drugs is of paramount importance. Different anesthesia methods and drugs possess their own merits and demerits. If the dosage can be precisely regulated, it can more effectively mitigate patients’ pain during treatment and promote recovery (24). These findings imply that the rational selection and application of anesthetic drugs and methods can significantly enhance the postoperative management of oncology patients. The development of anesthesia monitoring technology is of great significance in the postoperative management of oncology patients. Circulating biomarkers can be used to monitor cancer progression and postoperative recovery, such as markers related to the degree of malignancy of cancer cells, formation of the tumor microenvironment, and early metastasis, such as changes in the level of circulating tumor DNA (ctDNA) that can provide an early warning of tumor recurrence 3–6 months in advance for an individualized window of intervention (25), as well as markers related to systemic inflammation, immunosuppression, cognitive dysfunction, and pain management,. Providing new perspectives for perioperative management (26). In surgery for patients with cerebellar ataxia, neuropathy, and vestibular apraxia syndrome (CANVAS), specific anesthetic techniques and advanced hemodynamic monitoring can help prevent postoperative morbidity and optimize patient recovery. The anesthetic challenges of such patients can be better addressed through multidisciplinary discussions and careful perioperative planning (27). Optimization of anesthesia can significantly contribute to postoperative recovery in oncology patients. In elderly patients with gastrointestinal tumors, the combination of general anesthesia and TAP block results in reduced opioid dosage, improved pain control, better sleep quality, and a lower incidence of sleep disorders. The patients in this group in the study had an increased percentage of postoperative rapid eye movement sleep, fewer awakenings, and lower PSQI scores (*p* < 0.05) (28). This sleep improvement indirectly enhanced immune function and shortened the mean hospital stay by 2.3 days (*p* < 0.01) (29).

In pediatric tonsillectomy, the implementation of opioid-free anesthesia and analgesic regimen improves pain scores within 48 h after surgery. Opioids inhibit NK cell activity through mu receptor activation and affect the recovery of immune function in the postoperative period (30), improves the quality of sleep, shortens the time to the first meal, reduces the incidence of nausea and vomiting and insufficient analgesia, and improves the satisfaction of caregivers. It was shown that pain scores in the intervention group were significantly lower than the control group at 6, 12, and 24 h post-surgery (all *p* < 0.01), and remained lower at 48 h (31).

## 6 Historical evolution of the effect of anesthesia modalities and drugs on the postoperative period in oncology patients

### 6.1 Historical development of anesthetic management of oncology patients

The management of anesthesia in oncology patients has evolved over time. In the early days, anesthesia was mainly concerned with the patient’s painlessness during surgery, and as the understanding of oncology deepened, there was a gradual realization that anesthesia may have an impact on the prognosis of oncology patients. Modern studies have shown that opioids such as morphine may weaken the anti-tumor immune response by inhibiting NK cell activity (by 15–30%) and T lymphocyte proliferation (31). For example, for anesthesia of patients with central airway tumors, earlier the main focus was on airway obstruction and oxygenation, but nowadays there is a greater emphasis on comprehensive management, including response to emergencies such as tumor hemorrhage, pneumothorax, and cardiac arrest (32). Meanwhile, the choice of anesthetic drugs is optimized, such as giving priority to the use of analgesics with less immune impact, such as tramadol, to protect the recovery of postoperative immune function (33).

The understanding of the relationship between anesthesia-related factors and the prognosis of patients with oncology has been evolving in different periods of time. In the past, it was thought that certain anesthesia modalities might increase the risk of tumor recurrence, but as research has progressed, the reality has been found to be more complex. For example, a historical cohort study of patients with esophageal cancer showed no significant connection between anesthesia-related factors and postoperative acute kidney injury except for epidural anesthesia. It is worth noting that epidural anesthesia significantly reduces intraoperative systemic opioid dosage (62% reduction in morphine equivalents), which indirectly reduces immune suppression (34), and epidural anesthesia is potentially protective against early postoperative acute kidney injury (35).

### 6.2 Evolution of the administration of anesthetic drugs in the postoperative period in oncology patients

Administration of anesthetic drugs in postoperative oncology patients continues to evolve. In the past, opioids were the primary choice for postoperative analgesia, but in recent years their side effects have come under scrutiny. For example, postoperative opioid use has been correlated with the formation of clinically meaningful pancreatic fistulas post-distal pancreatectomy, with a 13% increase in the risk of pancreatic fistulae for each increase in total morphine equivalents of approximately 100 mg (advantage ratio is 1.13, confidence interval: 1.01–1.27, *p* = 0.035) (36).

As the understanding of anesthesia medications increases, novel medications and dosing regimens continue to emerge. In ovarian cancer surgery, implementation of an accelerated recovery surgery (ERAS) protocol resulted in a decrease in opioid use in patients within 24 h postoperatively (74.0 MME preoperative ERAS group vs. 25.8 MME ERAS group, *p* = 0.002), as well as a 55% decrease in opioid prescribing within 3 months of discharge (1101.4 MME preoperative ERAS group vs. 492.1 MME ERAS group, *p* < 0.001) (37). Breakthrough programs integrating targeted interventions with intestinal flora (e.g., oral administration of Mycobacterium fragilis DSM 2471) accelerated morphine metabolism through expression of *β*-glucuronidase (serum concentration ↓41%, *p* = 0.006), which led to a further reduction in morphine dosage in the ERAS group to 16.3 MME at 24 h postoperatively (*Δ* = -37%, *p* = 0.003) (38).

### 6.3 Historical changes in anesthetic techniques in oncologic surgery

Anesthesia techniques continue to advance in oncologic surgery. Early anesthesia techniques for tumor surgery were relatively simple, and with the development of medicine, a variety of advanced techniques have emerged. For example, in brain tumor surgery, the application of magnetic resonance imaging (MRI) technology has prolonged the operation and anesthesia time, but it has increased the tumor resection rate and improved the prognosis of patients. Studies have shown that patients using MRI technology-assisted surgery have smaller postoperative tumor volumes, higher tumor resection rates, and lower overall complication rates (*p* < 0.05) (39). Intraoperative 3D ultrasound-MRI fusion navigation technology increased the rate of total glioma resection to 92.3% (76.5% in the conventional group, *p* = 0.004) and reduced the rate of functional zone misdissection to 1.8% (7.4% in the conventional group, *p* = 0.009) (40).

Regional anesthesia techniques are also evolving. In limb surgery, regional anesthesia has gradually evolved from traditional methods to ultrasound-guided techniques, improving safety and effectiveness and reducing postoperative pain. At the same time, pharmacological advances in local anesthetic drugs and adjuvant medications have contributed to the development of regional anesthesia (41).

## 7 Effects of anesthetic drugs on tumor patients

### 7.1 Current standards and guidelines for the management of anesthesia in oncology patients

A series of standards and guidelines have been developed for the management of anesthesia in oncology patients. For example, the multimodal perioperative management (mPOM) guidelines for patients with colorectal and pancreatic cancers, which meet the S3 guideline requirements set by the German Association of Scientific Medical Specialties (AWMF), were developed through a process of systematic literature search and evidence quality assessment. It explicitly recommends limiting the use of potent *μ*-receptor agonists such as morphine, as their inhibition of NK cell activity (by 15–30%) may affect antitumor immune defense (42) Meta-analytic results demonstrate that mPOM decreases the complication rate in pancreatic and colorectal resections, with a risk difference (RD) of 0.96 (95% CI: 0.92–0.99) and risk ratio (RR) of 0.66 (95% CI: 0.54–0.80) and shorter hospital stay (43).

Guidelines for anesthesia management focus on different tumor types. For adrenal tumors, adequate hormonal diagnosis is required to identify patients with hormonal overdose, imaging tests such as CT, MRI and FDG - PET / CT are used to assess the degree of tumor malignancy, and surgery is the mainstay of treatment, with adjuvant postoperative therapy individualized as appropriate (44).

### 7.2 Effect of anesthesia modality and drugs on postoperative survival of oncology patients

Numerous studies have been conducted on the impacts of anesthesia modalities and medications on postoperative survival in oncology patients, but conclusions have been mixed. In thyroid cancer surgery, retrospective cohort Research has demonstrated that propofol anesthesia is linked to a lower recurrence rate compared with difluprednate anesthesia (risk ratio is0.38,95% confidence interval is between 0.25 and 0.56, *p* < 0.001), and this difference may be related to the reduction of opioid dosage by propofol-studies have shown that Inhibition of NK cell activity by morphine (15–30% reduction) impairs antitumor immune response (42) but there was no significant distinction in survival (*p* = 0.086) (45). Suggesting that the immunoprotective effect needs to be combined with long-term combination therapy (e.g., targeted therapy) to translate into a survival benefit (46).

In a cohort of 588 NSCLC surgical patients, independent predictors of shorter survival included at least 60-year-old patients, advanced tumor stage, and tumor size over3 cm, while factors associated with better survival were high BMI, mediastinal lymph node dissection, higher perioperative fentanyl dosage (>28.2 μg/kg), and high tumor grade (*p* < 0.05). Perioperative glucocorticoids were found to delay recurrence (*p* < 0.05) (47). Glucocorticoids protect short-term organ function by suppressing inflammatory factor storms, but may weaken T-cell anti-tumor responses in the long term, and the timing of their use needs to be weighed (47).

### 7.3 Effect of anesthesia modality and drugs influencing the postoperative quality of life among cancer patients

Anesthesia modalities and medications have a significant influence on postoperative quality of life in oncology patients. Based on radical thyroid cancer surgery, perioperative analgesia is improved by ultrasound-guided bilateral superficial cervical plexus block using ropivacaine or adjuvant medications, and this regimen significantly reduces intraoperative systemic opioid dosage (≥50% reduction in morphine equivalents), circumvents inhibition of NK cell activity by μ-receptor agonists, such as fentanyl (up to a 15–30% reduction), and reduces postoperative immune suppression-related fatigue (48) and post-operative quality of recovery. Compared with the general anesthesia-only group, the combined group had lower VAS scores at rest and during activity for 12 h postoperatively (*p* < 0.05) and better postoperative recovery indices (49).

In elderly patients undergoing major abdominal surgery, propofol anesthesia led to longer total sleep duration than sevoflurane anesthesia on the first postoperative night [median difference 29 min (95%CI4-53); *p* = 0.025], a difference that may be related to the significant reduction in intraoperative opioid dosage by propofol - the literature suggests that a morphine equivalent dose reduction of ≥40% attenuates its inhibition of NK cell activity (15–30% decrease), thereby reducing the disruption of sleep by immunosuppression-related fatigue (50). Compared with baseline, plasma orexin-A concentrations showed significant decreases at 1 h after anesthesia induction and 6 a.m. on POD1, with median reductions of 31.3 pg./mL (95% CI: −58.1 to −2.2, *p* = 0.033) and 29.8 pg./mL (95% CI: −58.3 to −2.3, *p* = 0.036), suggesting that propofol anesthesia has a positive effect on postoperative sleep quality. Has a positive effect (51).

While several studies suggest regional anesthesia reduces recurrence, conflicting data exist, particularly in non-immunogenic tumors. Many studies are retrospective, with potential confounding from surgical technique and adjuvant therapies.

## 8 Future prospects of research on anesthetic drugs in cancer patients

### 8.1 Prospects for anesthesia modalities and drugs in personalized postoperative treatment of oncology patients

Anesthesia modalities and drugs have a broad prospect for personalized postoperative treatment of tumor patients. In aged individuals with gastrointestinal neoplasms, combined general anesthesia and TAP block lowers opioid consumption according to the patient’s characteristics, alleviate postoperative pain, enhance the quality of sleep, and lower the occurrence of sleep-related disorders, which provides an idea of personalized anesthesia plan (28). Elevated postoperative serum S100A8/A9 protein levels in such patients were significantly correlated with tumor microenvironment pro-metastaticity (HR = 3.2, *p* = 0.009), while regional block anesthesia reduced its concentration by 41% (52). During neurosurgical procedures for brain tumors, Navigated Transcranial Magnetic Stimulation (nTMS) enables personalized brain mapping to guide surgical resection in patients who are not candidates for awake surgery. Studies have shown that nTMS -based mapping can reliably preoperatively risk-stratify patients, differentiate between tumors in true language-functioning areas and non-language-functioning areas, and help improve surgical safety and efficacy (53). An intraoperative real-time fluorescence navigation system based on nTMS data enables simultaneous visualization of tumor infiltration boundaries (92% sensitivity), improves the rate of total resection of high-grade gliomas by 28% (*p* = 0.002), and lower the danger of inadvertent hurt to normal brain tissue (54).

### 8.2 Directions for research on the long-term postoperative impacts of anesthesia modalities and drug therapies for postoperative care in oncology patients

Future in-depth studies are needed to explore the long-term effects of anesthesia modalities and medications on the postoperative outcome of oncology patients. There is currently no conclusive evidence regarding the impacts of distinct anesthesia modalities and medications on the long-term prognosis of oncology. For example, in a national cohort study Evaluating the effects of propofol TIVA compared with inhalation anesthesia on overall survival post cancer surgery, multivariate Cox proportional risk regression analyses showed that there was no significant correlation between the two types of anesthesia and overall survival post cancer surgery. (all *p* > 0.05), which may be related to differences in immunosuppression with opioid-adjuvant medications in different studies -e.g. morphine suppressed NK cell activity (15–30% reduction), whereas fentanyl equivalents > 25 μg/kg significantly reduced lymphocyte proliferation (55), but single-center studies showed higher overall survival in the TIVA group (pooled adjusted HR is 0.65, 95% CI is between 0.47 and 0.91, with *p* = 0.01), and the TIVA regimen attenuated T-lymphocyte functional suppression by reducing inhalation anesthetics and opioid dosage (≥40% reduction in morphine equivalents) (1.8-fold accelerated immune recovery shown in the literature), which may explain the single-center survival benefit (56), whereas the multicenter study showed no significant difference (pooled adjusted HR: 1.05, 95% CI: 0.82–1.33, *p* = 0.71) (57).

More prospective studies are still needed on the relationship between anesthesia modalities and postoperative delirium and long-term survival in older patients undergoing cancer surgery. Although there are retrospective studies suggesting that propofol-based intravenous anesthesia shows a potential link to extended survival, there is still an absence of prospective studies to confirm this association (58). In the future, studies on circulating tumor cell dynamics monitoring technology can be included to quantify the immediate effect of anesthesia on tumor dissemination by capturing the change in CTC counts in real time at 7 days postoperatively via microfluidic microarrays (>8/7.5 mL predicted a 35% decrease in 5-year survival, *p* < 0.001) (59).

Laboratory researches have indicated that volatile anesthetics like isoflurane and sevoflurane are capable of enhancing tumor cell proliferation, invasion and metastasis by activating pro-cancer signaling pathways such as HIF-1α and PI3K/Akt/mTOR at clinical concentrations (60, 61). For example, sevoflurane can down-regulate the toxicity of natural killer cells (NK cells) and weaken the body’s anti-tumor immune response (61). However, clinical findings are contradictory. Large retrospective analyses (e.g., Japanese study of GI tumors *n* = 196,303) showed no significant disparity in survival both volatile anesthesia and propofol-induced total intravenous anesthesia (TIVA), and randomized controlled trials (RCTs), such as CAN-study (*n* = 1,670), did not confirm an increased risk of recurrence.19 This discrepancy between laboratory and clinical findings may stem from the fact that sevoflurane has been shown to enhance perioperative tumor invasiveness and metastasis, and that the risk of recurrence is not as great as it seems. Differences may stem from the interference of multiple confounding factors (e.g., surgical trauma, patient immune status) in the perioperative period.

Propofol activates tumor cell apoptotic pathways (e.g., caspase cascade), inhibits migration (via upregulation of E-cadherin), and reduces chemoresistance (e.g., enhances cisplatin sensitivity) (62). Reduce release of pro-inflammatory factors (e.g., NF-κB) and protect T-cell function (61). Peritumoral infiltration of local anesthetics (e.g., lidocaine, ropivacaine) demonstrates significant clinical value: direct induction of iron death/apoptosis in tumor cells (via mitochondrial toxicity and inhibition of the PI3K/Akt pathway), and indirectly enhancement of anti-tumor immunity (e.g., protection of NK cell activity, promotion of dendritic cell-T cell response) (60, 63). Multicenter RCT in India (*n* ≈ 1,600) confirmed that intraoperative peritumoral infiltration of lidocaine (0.5%) in breast cancer increased 5-year disease-free survival from 83 to 87% (HR = 0.74) and overall survival from 86 to 90% (HR = 0.71) (62). General anesthesia/surgery induces monocytes to express pro-carcinogenic genes (e.g., MDM2) and reduces their anti-tumor capacity. Regional anesthesia, although theoretically reducing surgical stress and protecting T-cell function, has not been shown in RCTs to improve prognosis in breast or colorectal cancer (64).

### 8.3 Innovative strategies for anesthesia modalities and drug therapies for postoperative care in oncology patients

Innovative strategies are emerging in the postoperative management of oncology patients. In lung cancer surgery, optimizing anesthesia strategies based on patient histology, chemotherapy, radiotherapy, and epidural anesthesia can improve patient prognosis. For example, patients with lung adenocarcinoma require higher dosage of rocuronium bromide and midazolam in television-assisted thoracic surgery (VATS) and have faster postoperative recovery, suggesting that the dosage of anesthesia drugs should be adjusted according to the different types of lung cancer (65).

In terms of Postoperative pain control in patients with oral cancer, a multimodal approach, combined with adjuvant medications such as gabapentin and personalized pain management strategies, can improve pain management outcomes. Meanwhile, the application of nonpharmacologic interventions also provides new ways for pain management (66).

## 9 Controversial points on the postoperative effects of anesthesia modalities and drugs on oncology patients

The influence of anesthesia approach on the likelihood of postoperative recurrence in oncology patients has been a subject of dispute. Several studies have proposed that regional anesthesia could lower the risk of cancer recurrence, with the central mechanism being that regional anesthesia results in a notable decrease in the dosage of systemic opioids.(≥50% reduction in morphine equivalents) and circumvents the dose-dependent inhibition of NK cell activity by conventional opioids (e.g., morphine) (15–30% reduction) (67), a meta-analysis that included a total of 24,724 patients with cancer in 32 studies showed that regional anesthesia (RA) as a single modality or in conjunction with general anesthesia (GA) was linked to a significant lowering of cancer recurrence risk relative to GA alone (OR = 0.82,95% CI = 0.72–0.94, *p* < 0.01), and this protective effect was more pronounced in more immunogenic tumors (e.g., melanoma) due to the maintenance of NK-cell function to augment the efficiency of tumor-cell clearance (68), and in a subgroup analysis of prostate cancer patients Similar results were found (OR = 0.71,95% CI is between 0.51 and 0.98, *p* = 0.04) (64).

Nonetheless, some studies reach different conclusions. In a retrospective cohort study on surgery of oral cancer comparing propofol-based total intravenous anesthesia versus sevoflurane inhalation anesthesia, no significant disparity was observed between the groups in terms of overall survival and recurrence-free survival.(HR for overall survival was 1.10 in the sevoflurane group and 1.11 in the recurrence-free survival HR in the multivariate Cox analysis) (69).

Postoperative immunosuppression by anesthetic drugs in oncology patients is controversial. Opioids, for example, are widely used in general anesthesia, but their relationship with postoperative immunosuppression is unclear. A randomized controlled trial was designed to investigate the effect of analgesic techniques on opioid-induced immune perturbations and the feasibility of neutrophil-to-lymphocyte ratio (NLR) as an indicator of opioid-induced immune changes. The results showed that patients who received ultrasound-guided paravertebral block (PVB) combined with sufentanil patient-controlled intravenous analgesia (PCIA) had lower sufentanil consumption, lower NLR values (*p* < 0.0001). At 24 and 48 h after surgery, CD4/CD8 ratios were statistically higher (*p* < 0.05) when compared with those who received sufentanil-only PCIA, suggesting that opioid-sparing strategies may modulate postoperative immune suppression, and NLR can be utilized as a trustworthy metric for evaluating opioid-associated immune suppression (70). Changes in NLR values may reflect opioid-induced disorders of the immune system, as the literature suggests that opioids disrupt T-lymphocyte function via the *μ*-receptor, which indirectly supports the clinical significance of NLR as a marker of immunosuppression (71).

However, it has also been suggested that the results of current studies on immunosuppression by anesthetic drugs are inconsistent, and further investigations are required to elucidate the specific mechanisms and magnitude of their effects.

The influence of anesthetic management on the long-term postoperative prognosis of oncology patients is inconclusive. Some studies suggest that specific anesthetic management may influence prognosis, such as in breast cancer surgery, where perioperative infiltration with lidocaine may prolong The disease-free survival period and overall survival period of the patients, but regional anesthesia and propofol have not been shown to enhance oncologic outcomes (72).

However, other studies have reached different conclusions. When conducting research on patients with non-small cell lung cancer (NSCLC), although perioperative opioid and glucocorticoid exposure were found to be independent predictors of prognosis, the specific effects are more complex, e.g., perioperative fentanyl equivalents >28.2 μg/kg demonstrated a beneficial impact on overall survival, and high doses of fentanyl (>25 μg/kg), although inhibiting NK cell activity (20% decline), could be used to improve overall survival by suppressing the surgical stress-associated inflammatory storm has a short-term protective effect and may still impair immune surveillance in the long term (73), but increases the risk of postoperative pulmonary complications (*p* < 0.05) (47).

## 10 Critical comparison and methodological considerations

Despite a growing body of literature exploring the impact of anesthetic modalities and drugs on postoperative outcomes in cancer patients, significant heterogeneity and conflicting findings persist. A central controversy lies in whether total intravenous anesthesia (TIVA) with propofol offers oncologic advantages over volatile anesthetics. While several retrospective studies and meta-analyses suggest that TIVA is associated with improved recurrence-free or overall survival—particularly in immunogenic tumors such as breast or lung cancer—large cohort studies and randomized controlled trials (RCTs) have failed to confirm these benefits. For instance, a nationwide cohort study from Korea found no significant difference in long-term survival between propofol-based TIVA and inhalation anesthesia after cancer surgery, contradicting earlier single-center reports. Similarly, the role of regional anesthesia (RA) in reducing cancer recurrence remains debated. Although some meta-analyses report a modest protective effect (OR ≈ 0.82), others, including recent RCTs, show no significant impact on recurrence or survival. These discrepancies may stem from differences in tumor type, surgical stress, opioid use, and patient immune status, all of which interact with anesthetic techniques in complex ways. Everal methodological issues contribute to the inconsistency in findings: Study design: The majority of available evidence comes from retrospective cohort studies, which are prone to selection bias, confounding by indication, and incomplete data on perioperative variables such as opioid dosage, surgical technique, or adjuvant therapy. Heterogeneity in outcomes: Definitions of recurrence, survival endpoints, and immune markers vary widely across studies, limiting comparability and meta-analytic synthesis. Lack of standardization: Anesthetic protocols, including drug selection, dosing, and combination with opioids or regional blocks, are often not standardized, making it difficult to isolate the effect of a single agent or modality. Small sample sizes: Many studies are underpowered to detect differences in long-term oncologic outcomes, especially when stratified by cancer type or stage. Short follow-up: Some studies report only short-term immune or recovery outcomes without linking them to long-term survival or recurrence.

## 11 Conclusion

This review focuses on the impact of perioperative anesthesia management strategies, including anesthesia drugs and anesthesia modalities, on postoperative regression in oncology patients. Available evidence suggests that anesthesia choices have multidimensional effects that significantly influence patients’ perioperative and long-term prognosis. Inappropriate anesthetic drug or modality choices may adversely affect the special mechanism of prognosis in patients with malignant tumors, potentially increasing the danger of tumor recurrence and metastasis (Table 1). Therefore, there is an urgent need to adopt an individualized and comprehensive decision-making pathway for the anesthesia regimen for oncology patients, with the aim of minimizing potential negative effects and maximizing clinical benefits. Given the centrality of surgical treatment in comprehensive oncology, postoperative recurrence constitutes a major threat to long-term patient survival, accounting for approximately 90% of tumor-related causes of death (74). The regulation of perioperative immune homeostasis is a key component affecting tumor recurrence. Based on this, future research should focus on in-depth exploration of: the biological association between anesthesia and tumor, the interaction mechanism during anesthesia, etc. Deepening the above research will help optimize the perioperative management strategy of tumor patients and provide a solid theoretical foundation and evidence-based basis for improving their long-term survival rate.

## Abbreviations

AKI: Acute Kidney Injury
AWMF: Association of Scientific Medical Societies
CANVAS: Cerebellar Ataxia, Neuropathy, Vestibular Areflexia Syndrome
CD3+/CD4+: Cluster of Differentiation 3/4
CI: Confidence Interval
Cor: Cortisol
CTC: Circulating Tumor Cells
ctDNA: Circulating Tumor DNA
ERAS: Enhanced Recovery After Surgery
FDG-PET/CT: Fluorodeoxyglucose-Positron Emission Tomography/Computed Tomography
GA: General Anesthesia
GH: Growth Hormone
HIF-1α: Hypoxia-Inducible Factor-1α
HR: Hazard Ratio
IFN-*γ*: Interferon-γ
IL-1β/IL-4/IL-6: Interleukin-1β/4/6
MME: Morphine Milligram Equivalents
MRI: Magnetic Resonance Imaging
mPOM: Multimodal Perioperative Management
NK cell: Natural Killer Cells
NLR: Neutrophil-to-Lymphocyte Ratio
nTMS: Navigated Transcranial Magnetic Stimulation
OR: Odds Ratio
PCIA: Patient-Controlled Intravenous Analgesia
PEEP: Positive End-Expiratory Pressure
PR: Prevalence Ratio
PRL: Prolactin
PSQI: Pittsburgh Sleep Quality Index
PVB: Paravertebral Block
QoR-15: Quality of Recovery-15
RA: Regional Anesthesia
RCT: Randomized Controlled Trial
RD: Risk Difference
RR: Risk Ratio
STAT3: Signal Transducer and Activator of Transcription 3
TAP: Transversus Abdominis Plane
TGF-β1: Transforming Growth Factor-β1
Th1/Th2/Treg: T helper 1/2/Regulatory T cells
TIVA: Total Intravenous Anesthesia
VAS: Visual Analogue Scale
VATS: Video-Assisted Thoracoscopic Surgery
